# Decorrelative network architecture for robust electrocardiogram classification

**DOI:** 10.1016/j.patter.2025.101180

**Published:** 2025-01-22

**Authors:** Christopher Wiedeman, Ge Wang

## Main text

(Patterns *5*, 101116; December 13, 2024)

At the time of submission and peer review, Ge Wang was a member of the journal’s advisory board. The declaration of interests statement has now been updated to include this information.

